# Interaction between self-perceived disease control and self-management behaviours among Chinese middle-aged and older hypertensive patients: the role of subjective life expectancy

**DOI:** 10.1186/s12889-022-12990-8

**Published:** 2022-04-13

**Authors:** Jiao Lu, Linhui Liu, Jiaming Zheng, Zhongliang Zhou

**Affiliations:** 1 School of Public Policy and Administration, Xi’an Jiaotong University, Xi’an, Shaanxi China; 2 School of Management, Shanxi Medical University, Taiyuan, Shanxi China

**Keywords:** Self-management behaviours, Self-perceived disease control, SLE, Cross-lagged panel analysis, Mediation, Middle-aged and older hypertensive patients

## Abstract

**Background:**

One of the effective ways to control hypertension is long-term self-management, which is difficult to maintain. Therefore, understanding how people engage in the process of self-management behaviour change is necessary. In this study, we aimed to examine the dynamic relationship between self-perceived disease control and self-management behaviours in Chinese middle-aged and older hypertensive patients, namely, medication use, self-monitoring, physical activity, tobacco and alcohol avoidance, and to explore the mediating role of subjective life expectancy (SLE) on this relationship.

**Methods:**

Data were obtained from a nationally representative sample of 508 middle-aged and older hypertensive patients (aged 45+) from the 2013, 2015, and 2018 waves of the Chinese Longitudinal Healthy Longevity Survey. A cross-lagged panel model combined with mediation analysis was used to determine the dynamic relationship between self-perceived disease control and self-management behaviours and to clarify the mediating effect of SLE on this ascertained relationship.

**Results:**

Good self-perceived disease control subsequently predicted good medication use, self-monitoring and physical activity, and vice versa. Subjective life expectancy (SLE) partially mediated the prospective reciprocal relationships between self-perceived disease control and these self-management behaviours, which accounted for 37.11, 25.88, and 19.39% of the total effect of self-perceived disease control on medication use, self-monitoring and physical activity, respectively. These self-management behaviours had a significant and positive feedback effect on self-perceived disease control. However, neither the direct and indirect effects (via SLE) of self-perceived disease control on tobacco and alcohol avoidance were revealed.

**Conclusions:**

Positive feedback loops of present self-perceived disease control, future SLE and self-management behaviours (medication use, self-monitoring, and physical activity) help middle-aged and older hypertensive patients adhere to these behaviours but are useless for the avoidance of addictive behaviours. Interventions aimed at enhancing the effect perception of general self-management behaviours (e.g., medication use, self-monitoring and physical activity) on the present disease control perspective, and future lifespan perspective would be beneficial for the consistent self-management behaviours of middle-aged and older hypertensive patients. The utility of present disease control perception to these self-management behaviours was much higher than the utility of future expectations. Alternative stress relief strategies may be conducive to long-term changes in addictive behaviours.

**Supplementary Information:**

The online version contains supplementary material available at 10.1186/s12889-022-12990-8.

Hypertension has become a serious national disease in China [1], affecting more than 270 million adults [2], and more than 60% of them are middle-aged and older adults (≥45 years old) [3], which is a number that is on the rise [4]. This high prevalence is accompanied by high mortality, which accounts for more than half of the morbidity and mortality of cardiovascular diseases [1]. It is urgent to find an effective way to manage hypertension. Thus, self-management, which can be defined as the “active management by individuals of their treatment, symptoms, and lifestyle, etc., to better cope with disease” [5], has become the focus of health care for the increasing number of middle-aged and older hypertensive patients [6, 7]. Previous studies have also proven that self-management provides an opportunity for direct intervention at the individual level with the potential for favourable impacts on disease control and long-term health improvement [8–12]. Zhang et al. [13] assessed the clinical efficacy and health economics of self-management, traditional management and mobile apps for hypertension management, in which self-monitoring included home blood pressure monitoring, self-adjusting the antihypertensive regimen and setting a blood pressure warning value [13]. The results showed that self-management had a success rate of 83.4% and incurred the lowest cost of treatment [13]. Kaambwa et al. [14] compared the self-management of hypertension with usual care in terms of lifetime costs, quality adjusted life years and cost-effectiveness. When compared with usual care, self-management was more effective by 0.24 and resulted in 0.12 quality adjusted life years (QAL Ys) gained per patient for both men and women.

The concept of self-management originated from responsible stewardship of one’s health [15], which encouraged patients to be active participants in their day-to-day management of hypertension over the course of their illness [16]. An essential precondition for patient self-management of hypertension is patient empowerment [17], which is explained as an enabling process whereby health care professionals collaborate with patients to help them acquire knowledge and resources, and whose outcome is a patient with a greater ability to exercise control, manage their condition and make informed decisions [18]. However, the self-management of hypertension is boring and trivial and goes against the hedonic nature of human beings, which causes approximately 50% of patients to fail often when attempting to adhere to self-management behaviours [19, 20]. This failure inevitably undermines the effectiveness of treatments, leads to a deterioration of illness, and further increases preventable morbidity, members of hospitalization, burdens of diseases and health care costs [12]. Therefore, how to maintain patients’ adherence to self-management behaviours has become a key issue in the self-management of hypertension.

Because behavioural decisions guide subsequent behaviours later than individuals’ social-cognition factors do, researchers regularly apply social cognition theories to explore the mechanism of hypertension self-management adherence [21, 22]. However, these theories ignore the continuous, dynamic, and nonlinear process of health behaviour change in hypertension management [8]. Therefore, Leventhal et al. [23] proposed a dynamic framework based on the common-sense model of self-regulation (CSM), believing that illness-related perception and daily experience could lead to consistent self-management results. When an individual is diagnosed with hypertension, they first constructs a perception about disease control to change their behaviour associated with hypertension management. After that, the patients’ perception of disease control is further changed by monitoring and testing the performances and outcomes of self-management behaviours to see if they meet their expectations [10, 24, 25]. Then, self-management behaviour can become coherent and automatic through a two-way continuous update. This continuous change in present perception established from one’s practice can firmly encourage people to believe in and practice relevant ideas or things. Thus, we propose the first hypothesis that bidirectional causality between self-perceived disease control and self-management behaviour contributes to patients’ self-management adherence.

However, several systematic reviews have found that the relationship between the dimensions of disease-related perceptions and individuals’ self-management persistence behaviour is weak [9, 26]. Other factors may have influenced potential associations with subsequent self-management behaviours. The social cognitive theory argues that the closest predictor of target behaviour is motivation and that it creates a connection between cognition and related behaviour with an inherent “energy” force. Previous studies demonstrated that the estimates of subjective (self-rated) life expectancy (SLE), which reflected one’s future time perspective [27, 28], were seen as the primary motivational space in human self-regulation of their future behaviours [29] and may act as an inner motivation between patients’ self-perceived disease control and their self-management behaviours. SLE is an individual’s expectation regarding the perceived extent of his or her remaining years [28, 30] and has implications for apportioning health-related behaviour [31–34], especially in mid- and later life within an adjusted time frame [35]. Meanwhile, individuals always take their present genetic health, functional status and risk factors into account in their subjective evaluation of life expectancy [28, 36], and patients with a low sense of disease control perception tend to be less future oriented [37–39]. Therefore, we propose a second hypothesis: the mediator between self-perceived disease control and subsequent self-managed behaviour can be performed by SLE.

It is worth noting that the relationship between SLE and health-related behaviours is fuzzy. The vast majority of studies believed that adults who had low survival expectations were reluctant to engage in health-related behaviour and were even more likely to participate in a range of risky behaviours [7, 31, 32, 34, 40]. Others have argued that pessimistic expectations for survival may encourage people to live more carefully [41], and longer SLEs may lead to adverse self-management adherence behaviours [42]. In a study of African Americans, Irby-Shasanmi [43] reported that the relationships between longer SLE and risky behaviours on participants’ social networks are small but positive. Thus, we propose that the individual’s future self-management behaviour may also be interrupted by longer SLE, and that the mediating effect of SLE on self-perceived disease control and subsequent self-management behaviours is ambiguous.

Although some previous studies are valuable for identifying insight into the pathway among SLE, self-perceived disease control and self-management behaviours, the conclusions are difficult to evaluate the complex interactions among specific variables in the dynamics of self-management processes based on the cross sectional-based research design [21, 22]. At the same time, hypertensive patients’ self-management behaviours include medication use, self-monitoring, physical activity, tobacco and alcohol avoidance. The fuzzy effect of SLE on their self-management behaviours may be related to the differences in self-management behaviours, and this effect may be different in diverse cultural contexts. Therefore, the longitudinal data were analysed among middle-aged and older hypertensive patients in this study for two purposes: (i) to examine previously unexplored causality between self-perceived disease control and self-management behaviors and (ii) to assess whether SLE mediated the relationship between self-perceived disease control and self-management behaviours. Compared to current cross-sectional studies, this longitudinal study may further refine and verify Leventhal et al.’s [23] dynamic framework based on the common-sense model of self-regulation (CSM). Additionally, it might clarify the short-term and long-term cognitive mechanisms that persist in self-management behaviour by conducting a more thorough analysis of the relationship between individual illness related perception domains and self-management behaviour.

## Methods

### Participants

This study used secondary data analysis to test robust causal relationships. The data in our study came from the China Health and Retirement Longitudinal Survey (CHARLS), a dynamic cohort study with first-wave data collected in 2011 and three follow-up surveys in 2013, 2015, and 2018. The survey is one of the international health and pension survey series and is sponsored by the National Development Research Institute of Peking University, the Chinese Social Sciences Survey Center of Peking University, and the League Committee of Peking University. It recruits a representative sample of middle-aged and older adults over the age of 45 from 150 counties and 450 communities (villages) of 28 provinces, accounting for 85% of the total population in China. Ethical approval for all the CHARLS waves was granted from the Institutional Review Board at Peking University. In this study, all participants knew the purpose of the study and signed informed consent forms. The Institutional Review Board (IRB) approval number for the main household survey, including anthropometrics, is IRB00001052–11015; the IRB approval number for biomarker collection is IRB00001052–11014 [19, 20, 44–46].

Meanwhile, hypertension management has been behaviourally demanding and complex [47], and the core component of their care was considered self-management [20]. Given that the prevalence of hypertension and our ageing population are increasing, the impact of poor adherence to self-management behaviours on the health of the population is likely to become increasingly worse [9]. Therefore, we chose hypertensive patients as the participants in our study.

The current study used CHARLS’s last three waves of the data in 2013, 2015 and 2018 (recorded as T1, T2 and T3). The inclusion criteria were middle-aged and older hypertensive patients who participated in all three-wave investigations (2801 hypertensive patients). The exclusion criteria were (i) persons who lived with mental retardation and physical disabilities (429 hypertensive patients) and (ii) participants with missing data on the main variables (outcome variable and independent variable) (1864 hypertensive patients). Therefore, the final sample for analyses consisted of 508 respondents.

### Outcome variables

Self-management behaviours were the interaction of health behaviours, and patients and families engaging in the caring for a chronic condition was a related process [48]. Based on the relevant literature [12, 13, 49, 50] and CHARLS questionnaires, self-management behaviours in this study encompassed both pharmacological and nonpharmacological management behaviours, including medication use, self-monitoring, physical activity, and tobacco and alcohol avoidance.

#### Medication use

A single question, “Are you now taking any of the following medications to treat or control your hypertension?” was administered to assess participants’ medication use status. Each option of taking Chinese traditional medicine or modern Western was scored 1 when the answer was yes and 0 for none of the above, so higher scores indicated better medication use.

#### Self-monitoring

The question “During last year (last 12 months), how many times have you had a blood pressure examination?” was asked to inform the patients about their self-monitoring behaviour. The examination time was scored as 0, 1 for [1,6), 2 for [6,12), and ≥ 12 for 3. Higher scores indicated better self-monitoring behaviour.

#### Physical activity

The participation of physical activities (PA) were binary answers to the questions of whether an individual took vigorous physical activities (VPA), participated in moderate physical activities (MPA), or walked for at least 10 min continuously every week (WALK). CHARLS defined VPA as activities that made people breathe much harder than normal and might include heavy lifting, digging, ploughing, aerobics, fast bicycling, and cycling with a heavy load; MPA as activities that made individuals breathe somewhat harder than normal might include carrying light loads, bicycling at a regular pace, or mopping the floor; and WALK as walking that those individuals might engage in solely for recreation, sport, exercise, or leisure. PA in the database was divided into four levels according to exercise intensity, volume and time. The PA standard of level 1 was more than once a week with no less than 30 min of VPA each time, more than 3 times a week with no less than 30 min of MPA each time, or more than 5 times a week with no less than 30 min of WALK each time. Level 2 PA was no less than 30 min of MPA three times a week or no less than 30 min of WALK four or five times a week; Level 3 PA was for no less than 3 times a week with at least 30 min of WALK each time; Level 4 PA was not participating in physical exercise. Scores of 0–4 points were from level 4 PA to level 1 PA, and a higher score indicated better physical activity taking.

#### Tobacco avoidance

To understand patients’ tobacco avoidance status, we used questions of “Have you ever chewed tobacco, smoked a pipe, smoked self-rolled cigarettes, or smoked cigarettes/cigars?” and “Do you still have the habit, or have you totally quit?” to investigate the patients’ smoking history. The option of quitting or never smoking was scored as 1, and 0 was scored as still smoking.

#### Alcohol avoidance

The question “Did you drink any alcoholic beverages, such as beer, wine, or liquor in the past year? How often?” was used to understand the patients’ alcohol avoidance. The response of having ever drunk was scored as 0, and never drinking was scored for as 1.

### Independent variables

#### Self-perceived disease control

The self-perceived disease control of hypertensive patients was reported by the question, “Compared to when we interviewed you in R’s LAST IW MONTH, YEAR, is your condition better, about the same as it was then or worse?”. When the patient answered better, he or she scored 1, while he or she scored − 1 for worse and 0 for the same.

#### Mediating variable

Individual subjective life expectancy is usually gathered by the subjective probability of survival for a defined age or self-rated life expectancy. To calculate the subjective resident life (SLE), we refer to Spaenjers & Spira’s [51] study, and we calculate it as a proxy variable of SLE. The calculation formula was as follows:

1 $$\mathrm{Subjective}\ \mathrm{residual}\ \mathrm{life}=\mathrm{expected}\ \mathrm{age}\ \mathrm{at}\ \mathrm{death}-\mathrm{current}\ \mathrm{age}$$

2 $${\displaystyle \begin{array}{l}\mathrm{Expected}\ \mathrm{age}\ \mathrm{at}\ \mathrm{death}=\mathrm{average}\ \mathrm{life}\ \mathrm{expectancy}+\Big(\mathrm{target}\ \mathrm{age}-\mathrm{average}\ \mathrm{life}\ \\ {}\mathrm{under}\ \mathrm{the}\ \mathrm{same}\ \mathrm{probability}\Big)\end{array}}$$

The “current age” refers to the age of the respondents at the time of each survey.

The “average life expectancy” was determined by the China Life Insurance Mortality Table (2010–2013)^1^ (male = 79.5 years-old, female = 84.6 years-old).

The “average life under the same probability” was searched in the China Life Insurance Mortality Table (2010–2013) by the individuals’ ages and genders, and the same probability referred to an individual’s subjective survival probability (SPS), which was investigated by the CHARLS. The question was, “Suppose there are 5 options, where the lowest option represents the smallest chance, and the highest option represents the highest chance, on what option do you think is your chance of reaching the age of [...]?”. Therefore, the response options for this item were 1 = almost impossible, 2 = not very likely, 3 = maybe, 4 = very likely, 5 = almost certain, which correspond to the SPS of 0, 25, 50, 75 and 100%, respectively.

### Covariates

We included several covariates that were known to be associated with self-perceived disease control, and self-management behaviours were controlled in our statistical analyses to minimize the disturbing possibility of other variables and to maximize the parsimony of our analytic model [13, 52, 53]. The covariates included gender (0 = male, 1 = female), age (continuous variable), marital status (1 = married, 2 = unmarried, 3 = others (divorced and widowed)(reference)), Hukou types (0 = agricultural Hukou, 1 = nonagricultural Hukou), medical insurance (1 = urban employee medical insurance, 2 = urban and rural resident medical insurance, 3 = other medical insurance (reference)), education (1 = illiterate(reference), 2 = primary school and below, 3 = junior school and above), living arrangement (0 = living alone, 1 = living with others), comorbidity (Has the individual had other multimorbidity? (1 = Yes, 0 = No), these hypertension were dyslipidaemia (elevation of low density lipoprotein, triglycerides, and total cholesterol, or a low high density lipoprotein level); diabetes or high blood sugar; cancer or malignant tumour (excluding minor skin cancers); chronic lung diseases (such as chronic bronchitis); emphysema (excluding tumour, or cancer); liver disease (except fatty liver, tumour, and cancer); heart attack, coronary heart disease, angina, congestive heart failure, or other heart problems; stroke; kidney disease (except for tumour or cancer); stomach or other digestive disease (except for tumour or cancer); emotional, nervous, or psychiatric problems; memory-related disease; arthritis or rheumatism; asthma). Life satisfaction was assessed by a single question: “Please think about your life as a whole. How satisfied are you with it?” (1 = Not satisfied at all; 2 = Not very satisfied; 3 = Somewhat satisfied; 4 = Very satisfied; 5 = Completely satisfied). Social participation was assessed by a single question: “Have you participated in the following social activity in the past month?” for which there were 10 activities (interacted with a friend; played mahjong, chess, or cards, or went to community club; provided help to family, friends, or neighbours who did not live with you and did not pay you for the help; went to a sport, social, or another kind of club; took part in a community-related organization; did voluntary or charity work; cared for a sick or disabled adult who did not live with you and who did not pay you for the help; attended an educational or training course; stocked investment; and used the internet.). Participation in each activity was scored as 1; otherwise, it was scored as 0; and the range of the total score was 0–10 [54]. Self-rated health was evaluated by the question “What do you feel about your health status?” (1 = Very poor, 2 = Poor, 3 = Very good, 4 = Good, 5 = Fair). Medical intervention status was investigated by the question “Have you ever received any medical intervention?” The intervention items included blood pressure examination, weight control, physical exercise advice, diet advice, and smoking control. The answer “yes” was scored for 1, ranging from 0 to 5. Depression was determined by using a short form of the Center for Epidemiologic Studies Depression Scale (CES-D10) developed by Andresen et al. [55]. Lei et al. [56] tested the reliability and validity of CES-D10 by using CHARLS data to confirm the validity of CES-D10 through Chinese population studies, and CESD-10 covered a range of depressive symptomatology with an emphasis on current levels of depressive affect. Items were weighted by frequency of symptom occurrence in the last week using a 4-point Likert-type response format, and each item was rated from 0 (rarely or no time) to 3 (most or all the time). Individuals were divided into three groups based on the ranges identified by Andresen et al. [55]: 1 = depression (score ≥ 10), 0 = nondepression (score < 10). Activities of daily living (ADL) functional status was an index that indicated the individual functional status of middle-aged and elderly individuals when they dealt with ADL on their own. According to the international standard ADL index developed by Katz [57], ADL functional status contained six indices, which were the functional status of eating, dressing, transferring, bathing, using the toilet, and continence. Each item was independently completed with with 1 point; otherwise, it was 0, and the total score range was 0–6.

### Statistical approach

SPSS v26 (IBM Corp 2019) was used to test the demographic differences among self-management behaviours (independent-sample t test and one-way ANOVA) and the correlation among self-perceived disease control, SLE, and self-management behaviours (Pearson correlation analysis and multilinear test).

Two types of separate autoregressive cross-lagged models in Mplus v7.4 (Muthen & Muthen 1998–2015) were used to estimate the main hypotheses [58]. The first model examined the bidirectional association between self-perceived disease control and self-management behaviours, and the second model added SLE into the first model to test its role in bidirectional mediation. Both types of full models included stability paths within variables across time (i.e., autoregressive paths), concurrent associations among variables within each assessment wave, and associations among variables across time (i.e., cross-lagged paths). All analyses used the robust maximum likelihood (ML) estimator because the data were nonnormally distributed, which was determined by the K-S normality test. Model fit was evaluated by using criteria proposed by Hu and Bentler (1998; 1999), which used multiple fit indices comprising root mean square error of approximation (RMSEA) [59, 60], standardized root mean square residual (SRMR) and the ratio of chi-square to degrees of freedom (*χ*^*2*^/df). Model fit is good when RMSEA< 0.06, SRMR< 0.08, *χ*^*2*^/df < 3.

Furthermore, because the data distribution of variables was skewed, we used the bootstrapping method, which is an approach for implementing statistical tests and construct confidence intervals without the use of the traditional statistical assumption of normality used to test the statistical significance of the paths and to compute an estimation of the indirect effect with a 95% CI. The indirect effect was significant when the confidence interval did not include zero.

## Results

### Descriptive study

The distribution of the study variables measured in 2013, 2015, and 2018 are presented in Table 1. At baseline, 68.9% of the total respondents felt that their hypertension status had not changed; only 18.5% or 12.6% of the total felt that their hypertension was getting “better” or “worse”.

**Table 1:** Demographic characteristics of study participants (*n* = 508)

	2013(T1)		2015(T2)		2018(T3)	
	n/means	%/SD	n/means	%/SD	n/means	%/SD
**Gender**						
**Female**	307	60.43				
**Male**	201	39.57				
**Age**	62.73	7.22	64.73	7.22	67.73	7.22
**Marital status**						
**Married**	463	91.14	454	89.37	434	85.43
**Unmarried**	1	0.19	0	0.00	0	0.00
**Others (divorced and widowed)**	44	8.66	54	10.63	74	14.57
**Hukou types**						
**Agricultural Hukou**	374	73.62				
**Non-agricultural Hukou**	174	34.25				
**Education**						
**Illiterate**	92	18.11				
**Primary school and below**	116	22.83				
**Junior school and above**	300	59.06				
**Medical insurance**						
**Urban employee**	81	15.94				
**Urban and rural resident**	409	80.51				
**Other medical insurance**	18	3.54				
**Living arrangement**						
**Living alone**	312	61.41				
**Living with others**	196	38.59				
**Comorbidity**	2.43	1.44	2.75	1.45	3.88	1.89
**Life satisfaction**	2.84	0.74	2.62	0.73	2.77	0.79
**Social participation**	3.45	3.46	3.13	3.48	2.81	3.21
**Self-rated health status**	3.03	0.95	3.20	0.86	3.32	0.92
**Medical intervention status**	2.45	1.63	2.40	1.54	2.54	1.63
**Depression**	0.68	0.47	0.57	0.50	0.53	0.50
**Depression**	163	32.09	217	42.72	238	46.85
**Non-depression**	345	67.91	291	57.28	270	53.15
**ADL**	5.91	0.41	5.88	0.47	5.86	0.53
**Self-perceived disease control**			0.05	0.61	−0.07	0.65
**Getting worse**	64	12.60	82	16.14	128	25.20
**Same**	350	68.90	319	62.80	289	56.89
**Getting better**	94	18.50	107	21.06	91	17.91
**Subjective life expectancy**	17.93	9.56	15.93	8.64	13.74	8.43
**Medication use**	0.96	0.47	1.00	0.32	1.06	0.42
**Self-monitoring**	1.69	1.17	1.79	1.12	1.51	1.06
**Physical activity**	2.32	0.98	2.31	1.03	1.98	1.36
**Tobacco avoidance**						
**Smoking**	112	22.05	112	22.05	105	20.67
**Quit or never smoked**	396	77.95	396	77.95	403	79.33
**Alcohol avoidance**						
**Drinking**	150	29.53	128	25.20	137	26.97
**No drink**	358	70.47	380	74.80	371	73.03

### Variance analysis

Among the 508 respondents, the results of repeated-measures analysis of variance indicated that individuals’ self-perceived disease control (F = 2368.979, *p* < .001) and self-monitoring status (F = 2398.994, *p* < .001) decreased from 2013 to 2018, while individuals’ medication use (F = 6890.658, *p* < .001) and physical activity (F = 3012.893, *p* < .001) increased.

### Relevance analysis

The mean scores of the baseline cohort estimated from 2013 to 2018 were 0.06, 0.05, and − 0.07 for self-perceived disease control and 17.93, 15.93, and 13.74 for SLE, respectively. Pearson correlation tests for self-perceived disease control, SLE and self-management behaviours across the three waves are shown in Table 2 Additional file 1. Both within and across waves, self-perceived disease control, SLE and self-management behaviours were significantly and positively associated with each other.

### Reciprocal associations between self-perceived disease control and self-management behaviours

**Fig. 1:**
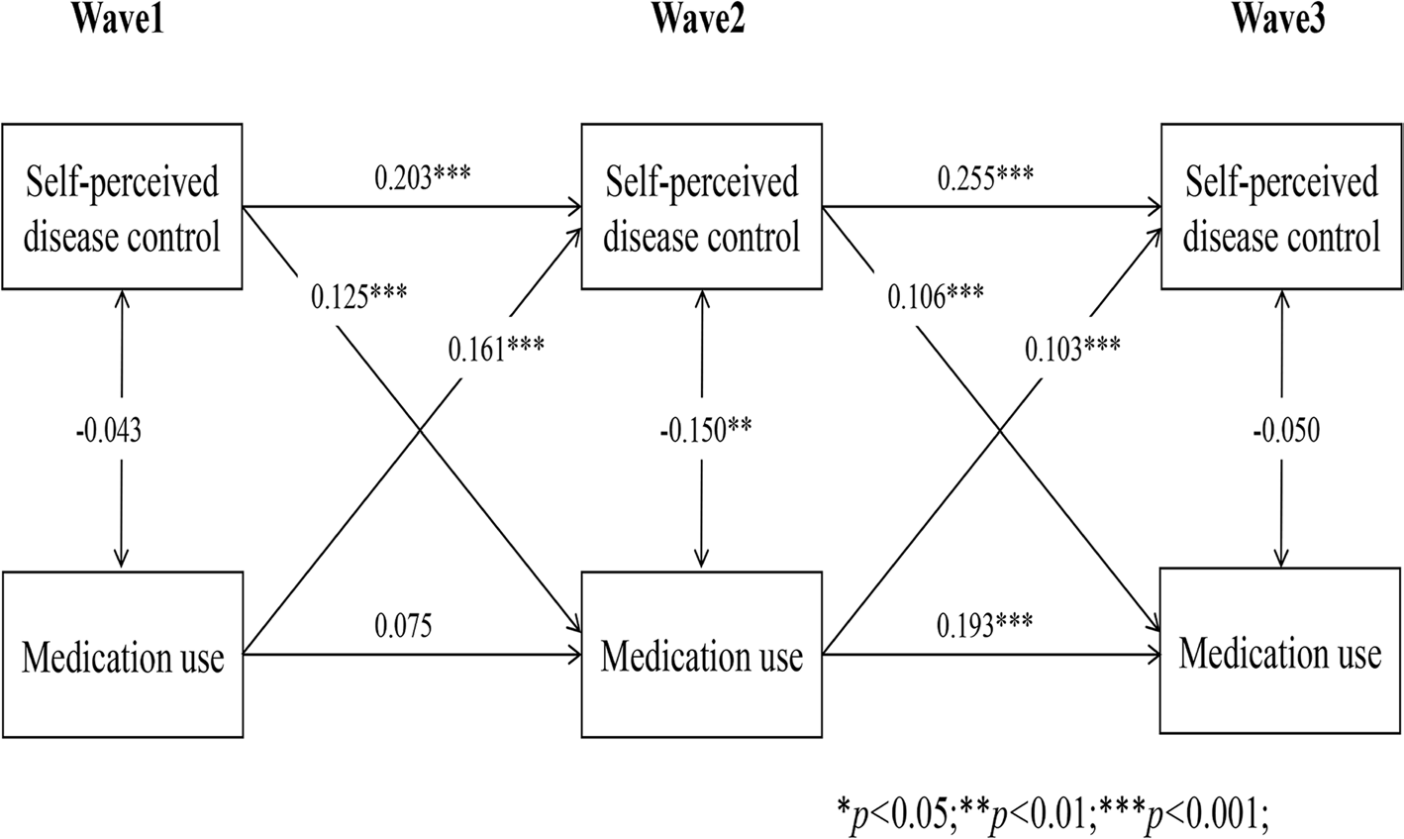
Autoregressive cross-lagged model showing the bidirectional associations between self-perceived disease control and medication use. Gender, age, marital status, Hukou types, medical insurance, education, living arrangement, comorbidity, life satisfaction, social participation, medical intervention status, depression, self-rated health status, and ADL were included as covariates in the model

**Fig. 2:**
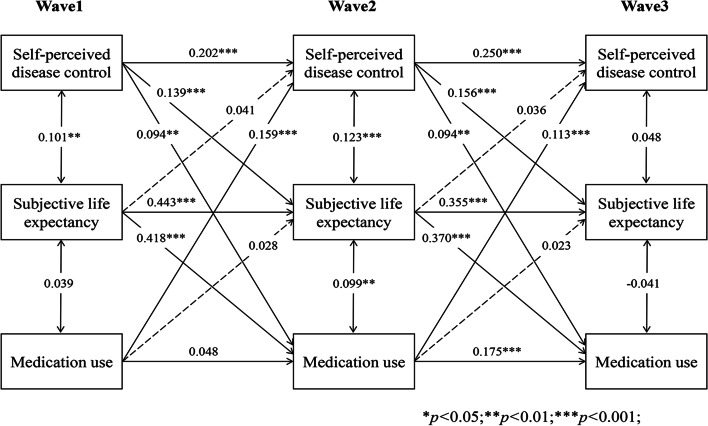
Autoregressive cross-lagged model showing the bidirectional associations between self-perceived disease control and medication use. Gender, age, marital status, Hukou types, medical insurance, education, living arrangement, comorbidity, life satisfaction, social participation, medical intervention status, depression, self-rated health status, and ADL were included as covariates in the model

**Fig. 3:**
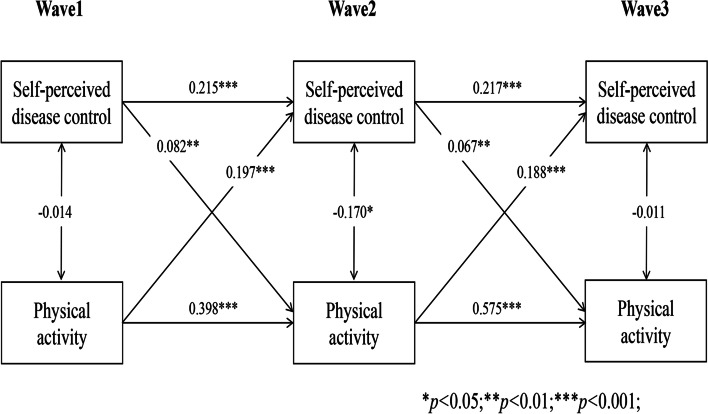
Autoregressive cross-lagged model showing the bidirectional associations between self-perceived disease control and medication use. Gender, age, marital status, Hukou types, medical insurance, education, living arrangement, comorbidity, life satisfaction, social participation, medical intervention status, depression, self-rated health status, and ADL were included as covariates in the model

**Fig. 4:**
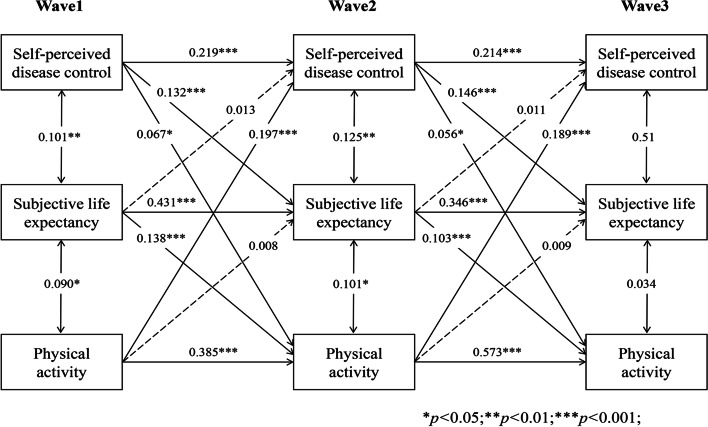
Autoregressive cross-lagged model showing the bidirectional associations between self-perceived disease control and medication use. Gender, age, marital status, Hukou types, medical insurance, education, living arrangement, comorbidity, life satisfaction, social participation, medical intervention status, depression, self-rated health status, and ADL were included as covariates in the model

**Fig. 5:**
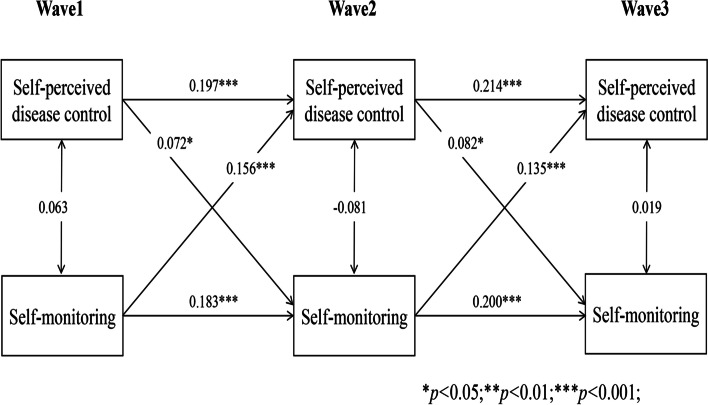
Autoregressive cross-lagged model showing the bidirectional associations between self-perceived disease control and medication use. Gender, age, marital status, Hukou types, medical insurance, education, living arrangement, comorbidity, life satisfaction, social participation, medical intervention status, depression, self-rated health status, and ADL were included as covariates in the model

Longitudinal cross-lagged models (Models 1–5) between self-perceived disease control and self-management behaviours (medication use, self-monitoring, physical activity, tobacco and alcohol avoidance) after controlling for covariates, with the autoregressive and concurrent estimates as well as cross-lagged paths, are illustrated in Figs. 1, 2, 3, 4 and 5. Models fitted the data adequately (RMSEA = 0.032 ~ 0.050, SRMR = 0.018 ~ 0.026, χ^2^/df = 1.52 ~ 2.25) (Supplementary Table 1 Additional file 2), and the 3-year cross-lagged effects of prior self-perceived disease control on medication use (β = 0.125, *p* < .001; β = 0.106, *p* < .001), self-monitoring (β = 0.072, *p* = .011; β = 0.082, *p* = .010), physical activity (β = 0.082, *p* = .006; β = 0.067, *p* = .006); and medication use (β = 0.161, *p* < .001; β = 0.103, *p* < .001), self-monitoring (β = 0.156, *p* < .001; β = 0.135, *p* < .001), physical activity (β = 0.197, *p* < .001; β = 0.188, *p* < .001) on subsequent self-perceived disease control were significant. No evidence for bidirectional causality was observed in the association between self-perceived disease control and tobacco/alcohol avoidance.

### Longitudinal indirect effect of SLE in the bidirectional association

**Fig. 6:**
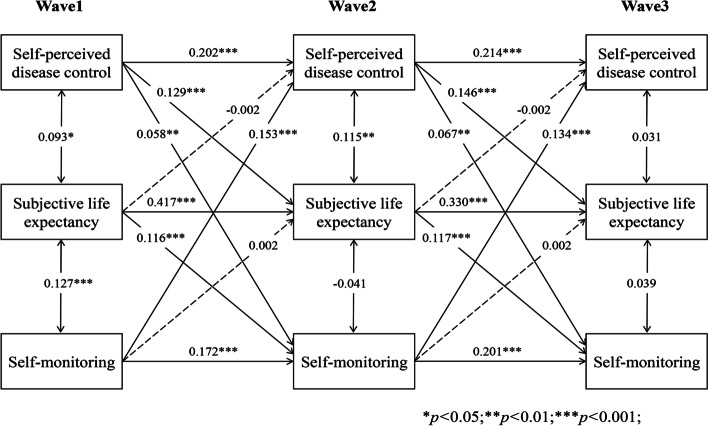
Autoregressive cross-lagged model showing the bidirectional associations between self-perceived disease control and medication use. Gender, age, marital status, Hukou types, medical insurance, education, living arrangement, comorbidity, life satisfaction, social participation, medical intervention status, depression, self-rated health status, and ADL were included as covariates in the model

**Fig. 7:**
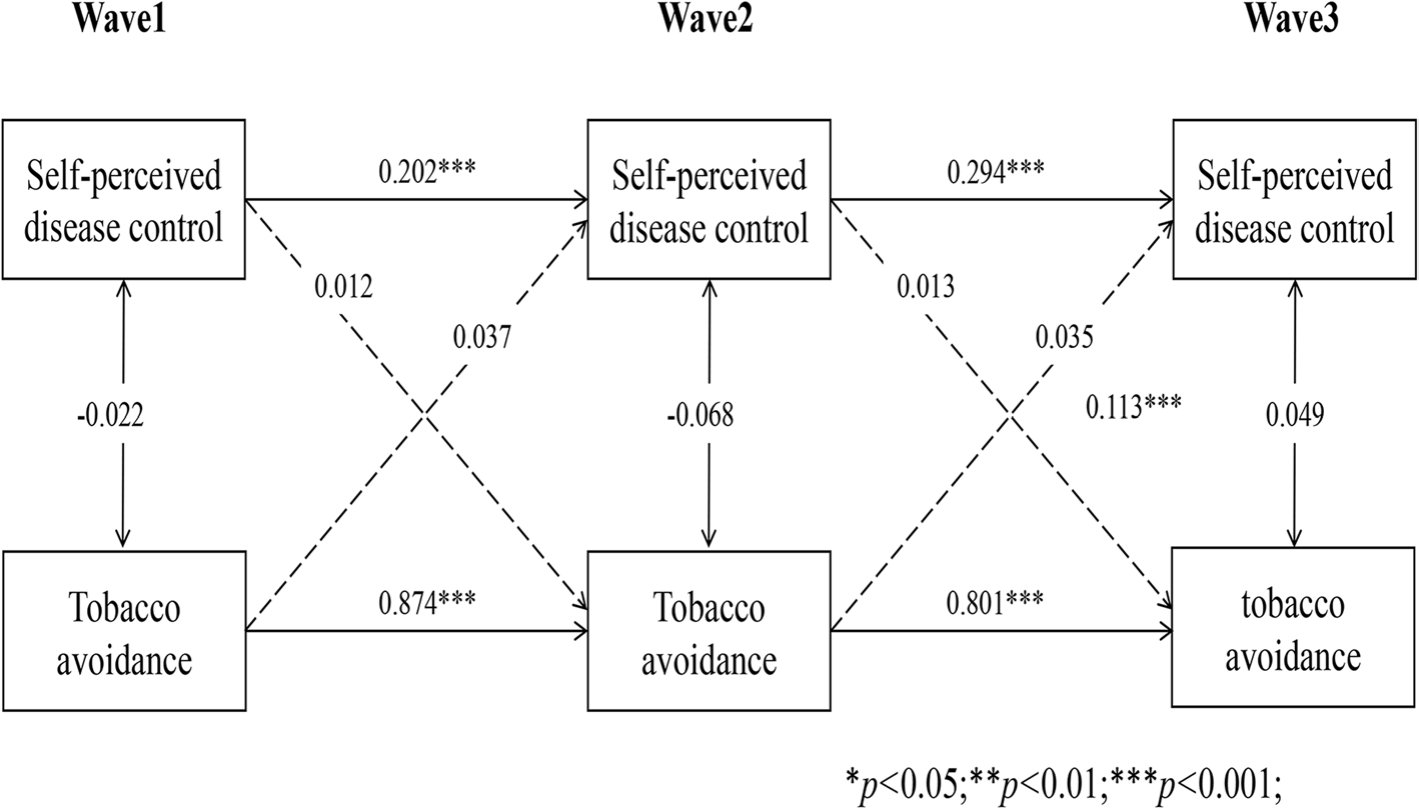
Autoregressive cross-lagged model showing the bidirectional associations between self-perceived disease control and medication use. Gender, age, marital status, Hukou types, medical insurance, education, living arrangement, comorbidity, life satisfaction, social participation, medical intervention status, depression, self-rated health status, and ADL were included as covariates in the model

**Fig. 8:**
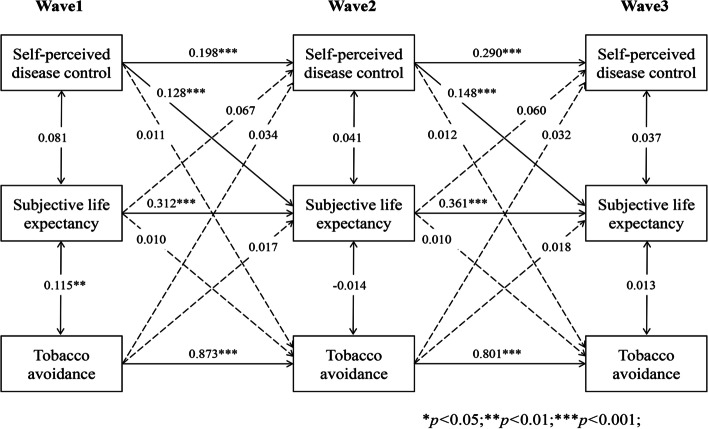
Autoregressive cross-lagged model showing the bidirectional associations between self-perceived disease control and medication use. Gender, age, marital status, Hukou types, medical insurance, education, living arrangement, comorbidity, life satisfaction, social participation, medical intervention status, depression, self-rated health status, and ADL were included as covariates in the model

**Fig. 9:**
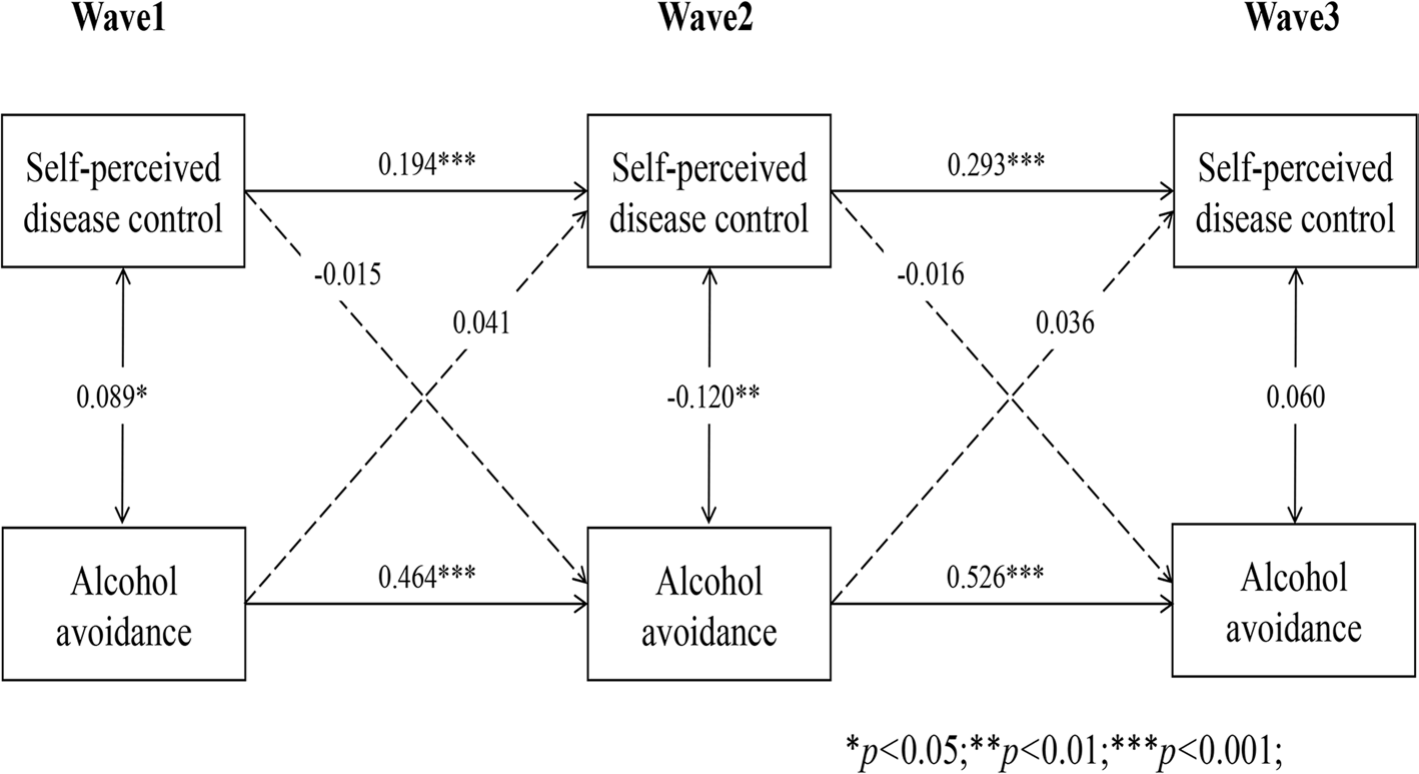
Autoregressive cross-lagged model showing the bidirectional associations between self-perceived disease control and medication use. Gender, age, marital status, Hukou types, medical insurance, education, living arrangement, comorbidity, life satisfaction, social participation, medical intervention status, depression, self-rated health status, and ADL were included as covariates in the model

**Fig. 10:**
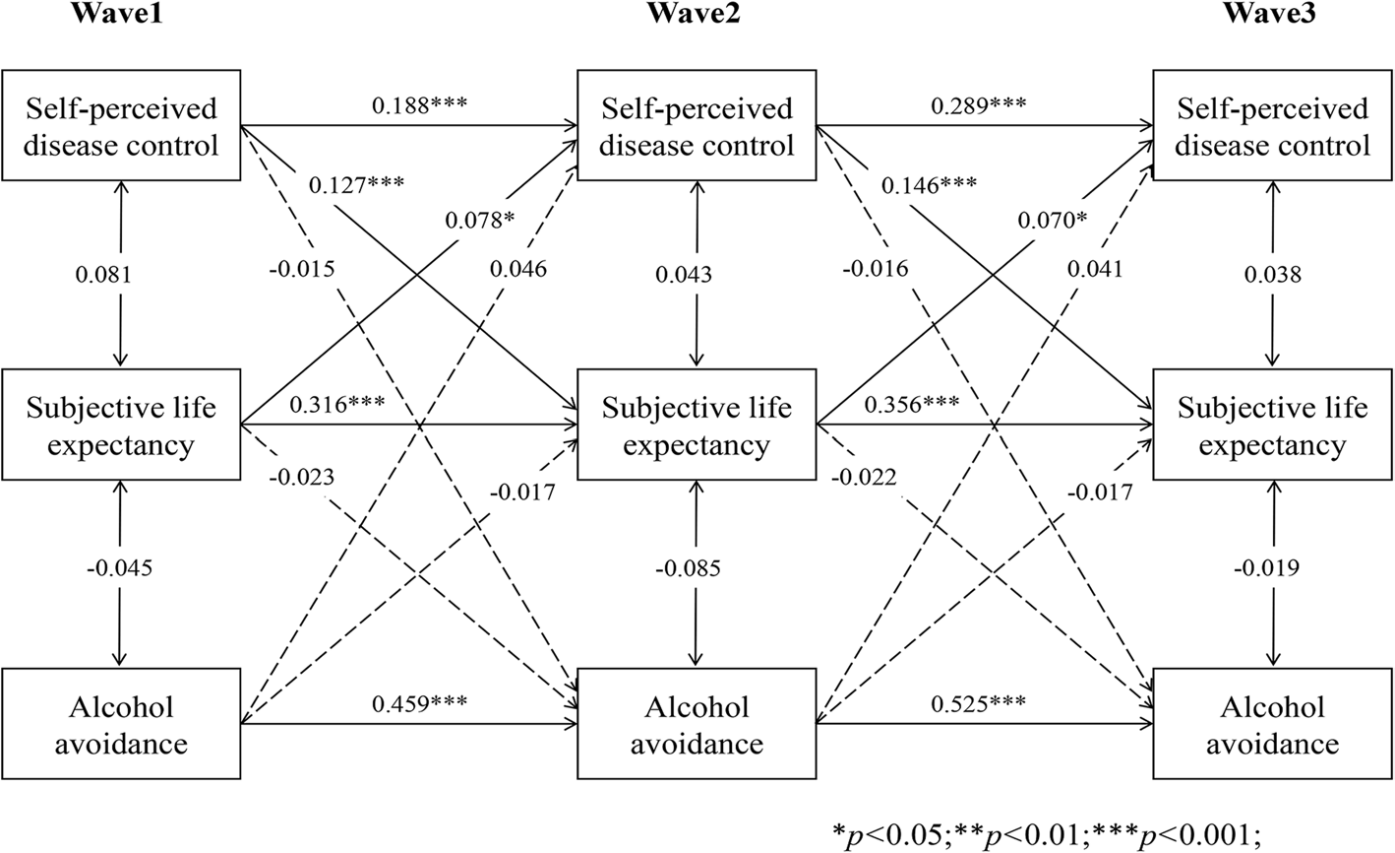
Autoregressive cross-lagged model showing the bidirectional associations between self-perceived disease control and medication use. Gender, age, marital status, Hukou types, medical insurance, education, living arrangement, comorbidity, life satisfaction, social participation, medical intervention status, depression, self-rated health status, and ADL were included as covariates in the model

As Figs. 6, 7, 8, 9 and 10 present, we tested the longitudinal mediation cross-lagged models by adding SLE as the mediator after adjusting for control variables. These models fit the data well (RMSEA = 0.042 ~ 0.058, SRMR = 0.026 ~ 0.037, χ^2^/df = 1.88 ~ 2.73) (Supplementary Table 2 Additional file 3), and the 3-year cross-lagged indirect effects revealed a significant indirect path from self-perceived disease control to medication use (β = 0.036, *p* < 0.001, 95% CI [0.025, 0.052]), self-monitoring (β = 0.019, *p* = 0.006, 95% CI [0.009, 0.033]), and physical activity (β = 0.022, *p* = 0.001, 95% CI [0.013, 0.035]) through SLE over time. The reverse cross-lagged direct effect but not the indirect effect from self-management behaviours (medication use, self-monitoring, and physical activity) to self-perceived disease control was shown over time. Meanwhile, these mediation models demonstrated that partial effects accounted for 37.11, 25.88, and 19.39% of the total effect of self-perceived disease control on medication use, self-monitoring and physical activity, respectively. Moreover, this mediation path was not observed between self-perceived disease control and tobacco/alcohol avoidance.

## Discussion

This study examined the reciprocal association between self-perceived disease control and self-management behaviours in middle-aged and older hypertensive patients and the underlying mechanism of SLE by explaining the process. The current longitudinal study documented the differentiated causal relationships among self-perceived disease control, SLE and various self-management behaviours through three waves over a 5-year period. These relationships are more complex than previously thought from cross-sectional research and have implications for adherence to middle-aged and older patients’ self-management behaviours [61, 62].

This study found a positive bidirectional association between self-perceived disease control and medication use, self-monitoring, and physical activity. It demonstrates that people will continue to implement self-management behaviours if they believe that these behaviours will help to prevent disease [63]. By validating and updating daily life, these experiences would pervade daily life. The prediction of illness-related perceptions of self-management behaviours for hypertension management has been confirmed by previous cross-sectional studies [49, 64, 65]. Qiu et al. [49] found that the acceptance of illness mediated the relationships between three types of health literacy and self-management and explored why health literacy and the acceptance of illness should be addressed when taking measures to improve patients’ self-management behaviour. Xie et al. [65] found that patient adherence may be improved by increasing patients’ self-management efficacy, using such means as patient empowerment, collaborative care, or enhanced patient-physician interactions. However, there was no clear evidence to prove the reverse prediction, and the important role of past behaviors for illness-related perceptions and consistent self-management behaviours has been certified by various studies [9, 26, 66]. In support of cognitive behavioural theories (e.g., theory of planned behaviour, cognitive behaviour therapy), past good experience was beneficial for individuals to simultaneously study self-management knowledge and perceive positive feelings in disease control [9, 24, 67, 68]. Enabling people to perform complex self-management activities might also enhance their self-perceived disease control [7, 69–72]. From the point of biological irritability and adaptability, as Gray’s reinforcement sensitivity theory (RST) pointed out, the perception of the disease control effect resulting from past self-management experiences might create rewards and punishments as stimulators. These stimuli can motivate behaviour approach responses and behavioural avoidance responses and help maintain self-management behaviours under repeated stimulation [68, 73].

Moreover, the positive regulatory role of SLE among self-perceived disease control and medication use, self-monitoring, and physical activity was separately identified in our study. This result indicates that when individuals perceive their disease control better, they tend to anticipate more positive and less negative outcomes over time — in turn, they tend to invest more self-regulatory effort (i.e., planning) and report high-level self-management behaviours. According to socioemotional selectivity theory [74, 75], individuals monitored their life expectancy based on their present health status and made different decisions in health self-regulation. An expansive time perspective was assumed to lead to a preference for goals aimed at optimizing the future (e.g., adopting a health behaviour), whereas a restricted future time perspective would be assumed to be related to a preference for emotionally meaningful goals and to be more concerned with a present payoff [31, 41, 76].

We also found that our results do not show reverse mediating effects, but that these behaviours feed back into self-perceived disease control, forming a potential feedback loop or spiral. This feedback loop may affect an individual’s adherence to medication use, self-monitoring and physical activity trajectory. Even though SLE has been shown to be associated with health behaviours, smoking status, alcohol status and physical activity through several studies [34, 35], this study further proved that SLE was a powerful predictor of behaviours.

Furthermore, we discover that in contrast to medication use, self-monitoring, and physical activity, the current study found no consistent patterns in self-perceived disease control and the avoidance of addictive behaviours (tobacco and alcohol avoidance). Self-perceived disease control can significantly influence SLE, but it does not affect tobacco and alcohol avoidance directly or indirectly through SLE. Additionally, addictive behaviours (e.g., smoking and drinking alcohol) have been regarded as the main risk factors for hypertension, but avoiding addictive behaviours (e.g., smoking and drinking alcohol) does not lead to better self-perceived disease control or SLE. High levels of social isolation and feelings of personal uselessness are more common among middle-aged and older individuals, leading to the further exacerbations of hypertension. Folkman and Lazarus (1986) defined “coping” as the cognitive and behavioural efforts to deal with (minimize, minimize, or endure) the internal and external demands of an interaction with the environment that a person determines may be burdensome to him or her, even beyond the resources available to him or her [77]. In addition to the need for social interaction, tobacco and alcohol are also good mechanisms for coping with stress in middle-aged and older hypertensive patients [20]. Tobacco and alcohol can help patients relax, feel good, relieve tension and anxiety, and pass the time [77]. Because tobacco and alcohol are stimuli that consistently release dopamine, the reward and reinforcement systems of the brain are activated by smoking and drinking. In turn, feelings of pleasure and satisfaction may drive individuals’ smoking and drinking alcohol automatically [78]. Thus, individuals are reluctant to give up their pleasure to quit smoking and drinking alcohol when medication use, physical exercise, and self-monitoring behaviours are available, even though they know that smoking and drinking alcohol are life-threatening. Additionally, this characteristic of middle-aged and older individuals of attaching importance to their present situation is consistent with the small mediating effect size of SLE in our study. The partial mediating effect of SLE on the prospective reciprocal relationships between self-perceived disease control and these self-management behaviours accounted for only 37.11, 25.88, and 19.39% of the total effect of self-perceived disease control on medication use, self-monitoring and physical activity, respectively. Moreover, consistent with previous studies [20, 50, 79, 80], our results proved that the significant effectiveness of medication therapy in disease control led to the strongest predictive effects of self-perceived disease control and SLE for medication use; middle-aged and older hypertensive patients are more likely to adhere to self-monitoring behaviour than they are to physical activity. The reason may be that medication use is the most timely and effective way to control hypertension among all self-management behaviours [81, 82], and self-monitoring behaviour is easy to adhere to because self-monitoring requires less effort than physical activity [83].

## Conclusion

This longitudinal study attempted to shed light on the underlying mechanisms linking self-perceived disease control and self-management behaviours among middle-aged and older hypertensive patients by using three waves of CHARLS data in 2013, 2015, and 2018, and the results were of great interest, as they had implications for the development of future intervention strategies for self-management behaviours. The present study indicates a bidirectional causality between self-perceived disease control and medication use, self-monitoring and physical activity, and the individual perception of “how long he or she will live” partially mediated the predtion of self-perceived disease control on these self-management behaviours (medication use, self-monitoring and physical activity) by forming a potential feedback loop or spiral. Individual adherence to medication use, self-monitoring and physical activity may be affected by these factors. However, this predictive relationship was not confirmed in tobacco and alcohol avoidance behaviours. Based on these findings, in establishing their health trajectories, the middle-aged and older hypertensive patients’ present self-perceived disease control and future lifespan expectations played important roles, which could uncover pathways to shape their self-management behaviours. It might be helpful to stress the notion of plasticity (i.e., hypertension can be largely controlled by self-management behaviours in the present, and gains in lifespan can be achieved in this way in the future). In particular, it might also encourage those with a limited time perspective to try to think about the short-term effect of self-management behaviours for disease control and the long-term effect on lifespan. However, because addictive behaviours (smoking and drinking alcohol) are difficult to affect by changing one’s self-perception of disease control and one’s future time perspective, patients might benefit from promoting the use of adaptive coping strategies (e.g., exercise and talking to family and friends) to actively cope with stress.

### Limitations

There are also some limitations to this study. First, autoregressive cross-lagged path analyses cannot separate the between-person effects from the within-person effects. Future studies should use the random intercept cross-lagged panel model focusing on within-person longitudinal associations to further test our results. Second, the study sample only comprised middle-aged and older hypertensive patients in China. It is unknown whether our findings will generalize to other cultural contexts and any other hypertensive patients. The results should be replicated in diverse social environments and patient populations. Third, there is a lack of analysis of other self-management behaviours (e.g., sleeping, dieting) due to the limitation of data. Other constructs of the illness belief domains or other mediators that have been well documented in empirical studies should also be examined further. Fourth, the complex associations between self-perceived disease control and self-management behaviours could be moderated by other factors (e.g., social support, regional medical level, personal health literacy, etc.). Future studies should elucidate the protective factors to strengthen self-perceived disease control or SLE to adherent self-management behaviours.

## Supplementary Information

**Additional file 1: Table 2.** Pearson correlation test for self-perceived disease control, subjective life expectancy and self-management behaviors.

**Additional file 2: Supplementary Table 1.** Fit index for longitudinal cross-lagged models (Model 1–5) between self-perceived disease control and self-management behaviors.

**Additional file 3: Supplementary Table 2.** Tested the longitudinal mediation cross-lagged models (Model 6–10) adding SLE as the mediator.

## Data Availability

The datasets generated and analysed during the current study are available in the [China Health and Retirement Longitudinal Survey] repository, [http://charls.pku.edu.cn/]. Public access to the database is open.

## Footnotes

1 The China Life Insurance Mortality Table (2010–2013) was released by the China Insurance Regulatory Commission in 2016. It collected data of 340 million policies and 1.85 million claims, covering 180 million people, ranking first in the world in terms of sample data. It accurately calculates the mortality probability of people of different ages and genders, and is one of the most authoritative standard for life expectancy measurement in China.
